# The Relationship between Extensively Drug-Resistant Tuberculosis and Multidrug-Resistant Gram-Negative Bacilli

**DOI:** 10.1371/journal.pone.0134998

**Published:** 2015-07-31

**Authors:** Jiang-nan Zhao, Xian-xin Zhang, Xiao-chun He, Guo-ru Yang, Xiao-qi Zhang, Huai-chen Li

**Affiliations:** 1 Department of Respiratory Medicine, Provincial Hospital Affiliated to Shandong University, Jinan, China; 2 Department of Respiratory Medicine, Shandong Provincial Chest Hospital, Jinan, China; 3 Department of Respiratory Medicine, Chest Specially Hospital of Weifang, Weifang, China; 4 Department of Tuberculosis Medicine, Chest Specially Hospital of Weifang, Weifang, China; University of Aveiro, PORTUGAL

## Abstract

**Objective:**

The relationship between extensively drug-resistant tuberculosis (XDR-TB) and multidrug-resistant Gram-negative bacilli (MDR-GNB) is unclear. Identification of the relationship between XDR-TB and MDR-GNB would have important implications for patient care.

**Methods:**

We conducted a retrospective study reviewing the records of patients admitted with a confirmed pulmonary TB from 2011 to 2014. To identify the relationship between XDR-TB and MDR-GNB, univariable comparison and multivariable logistic regression were performed.

**Results:**

Among 2962 pulmonary TB patients, 45(1.5%) patients had a diagnosis of XDR-TB. A total of 165 MDR-GNB strains were detected in 143 (4.8%) pulmonary TB patients. XDR-TB patients had a significantly higher occurrence of MDR-GNB than non-XDR-TB patients (24.4% vs. 4.5%; P<0.001). Age (OR 1.02, 95% CI 1.01–1.03), hypoalbuminemia (OR 1.48, 95% CI 1.18–1.85), chronic renal failure (OR 6.67, 95% CI 1.42–31.47), chronic hepatic insufficiency (OR 1.99, 95% CI 1.15–3.43), presence of XDR-TB (OR 6.56, 95% CI 1.61–26.69), and duration of TB diagnostic delay (OR 1.01, 95% CI 1.00–1.02) were the independent risk factors for MDR-GNB infection.

**Conclusions:**

Patients with XDR-TB have a significantly higher risk of being affected by MDR-GNB pathogen. The underlying mechanism association warrant further studies.

## Introduction

The extensively drug-resistant tuberculosis (XDR-TB) has severely threatened TB control worldwide [1,2]. According to World Health Organization, XDR-TB has been reported of an estimated 43,200 people by 100 countries in 2013 [3]. Patients who develop XDR-TB have subtle immune defects that could be vulnerable to other bacterial infection. The incidence of nosocomial infection caused by Gram-negative bacilli (GNB) has increased at an alarming rate [4,5]. The emergence of multidrug-resistance (MDR) among GNB organisms also increases deserving particular concern, because antimicrobial agents for MDR strains are often limited and inappropriate treatment will predispose these patients to an especially poor outcome, even death [4–6].

Considering that broad-spectrum antibiotics are frequently used in patients with XDR-TB [7–9] and that anti-TB drugs have antibacterial activity, patients with XDR-TB are susceptible to harboring antimicrobial resistance. In clinical practice, we realized that XDR-TB patients had a high occurrence of harboring MDR-GNB compared to non-XDR-TB patients. MDR-GNB in XDR-TB patients would be ominous. However, there remains a paucity of convincing data about the relationship between MDR-GNB and XDR-TB.

Although previous studies have identified risk factors for MDR-GNB [5,10,11], risk factors for harboring MDR-GNB among pulmonary TB patients have not been elucidated. The objectives of this study, therefore, were to determine the association between MDR-GNB and XDR-TB, and assess the risk factors for MDR-GNB among pulmonary TB patients.

## Methods

### Ethics statement

The study was approved by the Ethic Committee of Shandong Provincial Hospital, affiliated to Shandong University. Patient records were anonymized and de-identified prior to analysis.

### Study population and data collection

From January 1, 2011 to December 31, 2014, the retrospective cohort study included consecutive pulmonary TB patients aged ≥ 18 years admitted to Shandong Provincial Hospital, Shandong Chest Hospital and Chest Specially Hospital of Weifang, which elaborated a common research protocol. Patients with both drug susceptibility testing (DST) results and medical records available were included for further analysis. Patients infected by human immunodeficiency virus (HIV) were excluded in our study, since HIV-positive patients will be transferred to HIV specialized hospital immediately in China.

All patients’ information was routinely collected and recorded by trained research coordinators over the entire study period. We obtained the following information from medical records for all study patients: age, sex, occupation, history of close contact with a TB patient (defined as a household member or colleague with TB), TB treatment history, smoking history (more than 10 years smoking), excess alcohol consumption (more than 2 standard alcohol beverages per day), co-morbid conditions (according to the discharge diagnosis), central venous catheter, invasive mechanical ventilation, parenteral nutrition, laboratory results, chest radiography results, hospital length-of-stay (LOS), duration of TB diagnostic delay (the onset of pulmonary symptoms to the diagnosis of TB), and hospital discharge status (survive, dead). The results of identifying MDR-GNB collected after the diagnosis of XDR-TB were reviewed and analyzed.

To identify the risk factors for MDR-GNB, patients with harboring bacteria other than *Mycobacterium tuberculosis (MTB)* isolated during anti-TB treatment in hospitals were included. All enrolled patients were divided into two groups: TB patients with harboring MDR-GNB and those with harboring non-MDR-GNB. We compared two groups to determine the independent risk factors.

### Bacteriologic examinations

Specimen of the clinically suspicious infection site was sampled for culture. Infection categories in this study included pulmonary infection; gastrointestinal infection; cardiovascular system infection; central nervous system infection; urinary tract infection; reproductive tract infection; bone and joint infection; eye, ear, nose, throat, and mouth infection; skin and soft tissue infection; surgical wound infection; bacteremia; and systemic infection.

Expectorated sputum was collected into a sterile container. Microscopy by Gram staining was performed for presence of leucocytes, epithelial cells and organism morphotypes. Those containing greater than 25 leucocytes and fewer than 10 squamous epithelial cells per field were optimal specimens and further processed. The quality of specimens was evaluated based on Gram stain findings, followed by culture and susceptibility testing. Urine fluid was processed by quantitative culture with positive threshold of 10^5^ CFU/ml. Sputum, urine and other isolates harvested from infection sites were inoculated on proper culture media and then resubjected to further susceptibility testing on Muller-Hinton agar following the standard microbiologic methods according to the criteria of clinical laboratory standard institute (CLSI) guidelines [12]. Culture plates were reincubated for a further 24 h if there was no growth after overnight incubation or predominant morphotype seen in Gram smear had not yet been isolated. The following agents were tested: imipenem, meropenem, cefepime, ceftazidime, ceftriaxone, penicillin G, piperacillin, ticarcillin, ampicillin/sulbactam, aztreonam, ciprofloxacin, levofloxacin, moxifloxacin, gentamicin, amikacin.

Acid fast staining and culture for *MTB* on Lowenstein–Jensen medium after decontamination of sputum specimens were performed. Drug susceptibility testing (DST) was determined by means of the proportion method, with following concentrations of the drugs [13]: isoniazid (0.2 μg/mL), rifampicin (40 μg/mL), ethambutol (2.0 μg/mL), streptomycin (4.0 μg/mL), kanamycin (30 μg/mL), capreomycin (40 μg/mL), ofloxacin (2.0 μg/mL), levofloxacin (2.0 μg/mL), para-aminosalicylic acid (1.0 μg/mL). The *MTB* isolates were considered to be resistant if there was more than 1% growth on medium containing anti-TB drugs as compared with the growth on drug-free medium. Susceptibility testing to pyrazinamide and the remaining second line drugs are not routinely done. External quality assessment (EQA) was conducted regularly by TB National Reference Laboratory.

### Definitions

Infection was defined according to criteria proposed by the Centers for Disease Control and Prevention (CDC) [14]. Infection was developed after 48h of hospitalization that classified as nosocomial infection. GNB infection was defined on the basis of GNB organism isolated from cultures of tissue or fluid from the affected site with clinical signs and symptoms of infection.

According to CDC criteria, MDR strains were defined as those resistant to ≥ 1 agent in 3 or more of the following antimicrobial categories [5,14]: carbapenems (imipenem, meropenem), penicillins (piperacillin, ticarcillin, ampicillin/sulbactam), broad-spectrum cephalosporins (ceftazidime, cefepime), monobactams (aztreonam), aminoglycosides, and fluorquinolones. The following conditions were also considered as the presence of MDR strains [6,14]: (1) organisms with intrinsically resistance to the broadest-spectrum antimicrobial agents such as *Stenotrophomonas maltophilia*; (2) *Pseudomonas aeruginosa* resistant to at least three of the following antimicrobial groups: ceftazidime/cefepime, aminoglycosides, fluoroquinolones, carbapenems, and piperacillin; and (3) *Acinetobacter baumannii* resistant to all antimicrobial agents, or all except imipenem.

MDR-TB is defined as TB isolate resistant to at least both isoniazid and rifampicin. XDR-TB is defined as TB isolate resistant to both isoniazid and rifampicin, plus resistance to a fluoroquinolone and one of three injectable second-line drugs (kanamycin, capreomycin, or amikacin) [15].

### Statistical analysis

Continuous variables are summarized with mean and standard deviation (SD); categorical variables are summarized as proportions. In univariable analysis, student’s t-test is used to compare continuous variables, and Pearson’s *X* ^*2*^ test or Fisher’s exact test are used to compare categorical variables. To identify independent factors that are associated with MDR-GNB, multivariable logistic regression analysis is used. The odds ratios (OR), 95% confidence interval (CI) and P value for individual variables are obtained using a logistic regression model, and P<0.05 is considered to be statistically significant. To assess the discriminatory ability of the model, the *c* statistic are calculated, which represents the area under the receiver operating characteristic (ROC) curve, ranges from 0.5 (which indicates no better discrimination than chance) to 1.0 (perfect discrimination). The area under the curve (AUC) and its standard error (SE) are also obtained. Statistical analysis is performed using SPSS software, version 16.0.

## Results

### Demographic and clinical characteristics of XDR-TB patients

During the study period, 4157 patients aged ≥ 18 years with the diagnosis of pulmonary TB were recorded. Only 3184 patients had performed DST. Among these patients, results of DST and medical records were available for 2962 patients.

The valid DST results showed that 45 patients were classified as having XDR-TB, accounting for 1.5% of all patients. Demographic and clinical characteristics of XDR-TB patients were recorded in Table 1. Of the patients with XDR-TB, the mean age was 46 years (mean±SD, 45.6±17.1 years); 22 patients (48.9%) were male.

**Table 1:** Demographic and clinical characteristics of XDR-TB patients.

	Non-XDR-TB	XDR-TB	P value
	N = 2917(%)	N = 45(%)	
Sex male	1679(57.6)	22(48.9)	0.260
Age (years)	46.1±19.6	45.6±17.1	0.871
Smoking history	963(33.0)	21(46.7)	0.057
Excess alcohol consumption	453(15.5)	8(17.8)	0.837
TB re-treatment	553(19.0)	28(62.2)	<0.001
TB contact history	171(5.9)	5(11.1)	0.188
Cavity lesion	1883(64.6)	38(84.4)	0.007
Hypoalbuminemia	2018(69.2)	41(91.1)	0.002
Anemia	1204(41.3)	25(55.6)	0.067
Chronic renal failure	28(1.0)	1(2.2)	0.360
Chronic hepatic insufficiency	462(15.8)	3(6.7)	0.101
Extra-pulmonary TB	497(17.0)	11(24.4)	0.228
Chronic pulmonary disease^a^	209(7.2)	8(17.8)	0.015
Gastric ulcer	57(1.9)	1(3.0)	0.481
Cardio-cerebrovascular disease	137(4.7)	2(6.1)	0.667
Hypertension	169(5.8)	4(12.1)	0.123
Diabetes	460(15.8)	9(20.0)	0.536
CTD	51(1.7)	2(4.4)	0.192
Malignant	41(1.4)	0(0)	1.000
Nosocomial GNB infection	396(13.6)	15(33.3)	0.021
Caused by MDR-GNB	132(4.5)	11(24.4)	<0.001
Hospital LOS (days)	40.6±15.3	59.9±19.5	0.021
Duration of TB diagnostic delay (days)	44.1±22.5	57.8±26.7	0.022
Hospital mortality	215(7.4)	13(28.9)	<0.001

Abbreviation: XDR-TB, extensively drug-resistant tuberculosis; CTD, connective tissue disease; GNB: Gram-negative bacilli; MDR, multidrug-resistant; LOS, length of stay.

Note

^a^ Chronic pulmonary disease included chronic obstructive pulmonary disease, asthma, bronchiectasis.

Patients were divided into XDR-TB group and non-XDR-TB group. A significantly greater proportion of patients with XDR-TB had previous anti-TB treatment, cavity lesion, and hypoalbuminemia (P<0.05). Compared to non-XDR-TB patients, XDR-TB patients had higher occurrence of nosocomial GNB infection (33.3% vs. 13.6%; P = 0.021) and MDR-GNB infection (24.4% vs. 4.5%; P<0.001). Patients with XDR-TB had longer hospital LOS (days) (59.9±19.5 vs. 40.6±15.3; P = 0.021) and longer duration of TB diagnostic delay (days) (57.8±26.7 vs. 44.1±22.5; P = 0.022). They were also had a higher hospital mortality (28.9% vs. 7.4%; P<0.001).

### Results of microbiology

A total of 526 GNB strains were detected in 411 patients. Results of microbiology are shown in Table 2. The most frequently detected pathogen was *Klebsiella pneumoniae* (n = 150, 28.6%). A total of 165 MDR-GNB strains were detected in 143 patients. Of 143 patients, MDR organisms were isolated from sputum (28.7%), pleural effusion (7.0%), urine (34.2%), blood (11.9%), surgical wound (7.7%), bile fluid (2.1%), skin (6.3%), bone (1.4%), and ear (0.7%). Rates of MDR-GNB strains were as follows: MDR-*Enterobacter cloacae* (13.9%), MDR-*Klebsiella pneumoniae* (33.3%), MDR-*Escherichia coli* (9.7%), MDR-*Pseudomonas aeruginosa* (21.8%), MDR-*Acinetobacter baumannii* (7.9%), MDR-*Stenotrophomonas maltophilia* (1.8%), MDR-*Proteus mirabilis* (4.2%), MDR-*Klebsiella oxytoca* (3.0%) and MDR-*Serratia marcescens* (4.2%).

**Table 2:** GNB and MDR-GNB pathogens that cause nosocomial infection among pulmonary TB patients.

GNB pathogens	Total N = 526(%)	MDR N = 165(%)
*Enterobacter cloacae*	59(11.3)	23(13.9)
*Klebsiella pneumoniae*	150(28.6)	55(33.3)
*Klebsiella oxytoca*	18(3.4)	5(3.0)
*Escherichia coli*	54(10.3)	16(9.7)
*Pseudomonas aeruginosa*	98(18.7)	36(21.8)
*Acinetobacter baumannii*	44(8.4)	13(7.9)
*Stenotrophomonas maltophilia*	3(0.6)	3(1.8)
*Proteus mirabilis*	21(13.5)	7(4.2)
*Serratia marcescens*	24(3.4)	7(4.2)
Others^a^	55(10.5)	0

Abbreviation: GNB: Gram-negative bacilli; MDR, multidrug-resistant.

Note

^a^ Others included *Pseudomonas fluorescent* (5 cases), *Chryseomonas luteola* (3 cases), *Aeromonas hydrophila* (5 cases), *Acinetobacter lwoffi* (2 cases), *Citrobacter freundii* (3 cases), *Chryseobacterium indologenes* (4 cases), *Enterobacter aerogenes* (3 cases), *Shewanella putrefaciens* (3 cases), *Alcaligenes xylosoxidans* (5 cases), *Selenomonas dianae* (2 cases), *Serratia liquefaciens* (3 cases), *Pasteur's pneumotropica* (3 cases), *Haemophilus influenzae* (6 cases), *Burkholderia cepacia* (4 cases), *Morganella morganii* (1 case), *Enterobacter asburiae* (2 case), *Moraxella catarrhalis* (1 case).

### Characteristics of patients with MDR-GNB and risk factors for MDR-GNB

Baseline and demographic characteristics of patients with MDR-GNB and non-MDR-GNB are shown in Table 3. Of the 143 patients with MDR-GNB, the mean age was 53 years (mean±SD, 53.2±20.7 years); 68 patients (47.6%) were male. The mean hospital LOS (days) and duration of TB diagnostic delay were significantly longer in MDR-GNB patients than non-MDR-GNB patients. The overall hospital mortality rate of patients with MDR-GNB was 15.4% compared to 7.5% of patients with non-MDR-GNB (P = 0.016).

**Table 3:** Univariable and multivariable analysis of risk factors for MDR-GNB infection among pulmonary TB patients.

	Non-MDR-GNB	MDR-GNB	Univariable analysis		Multivariable analysis	
Variables	N = 268(%)	N = 143(%)	OR (95%CI)	P value	OR (95%CI)	P value
Age	45.23±19.15	53.22±20.71	1.02(1.01–1.03)	<0.001	1.02(1.01–1.03)	<0.001
Sex						
Male	94(35.1)	68(47.6)	Reference			
Female	174(64.9)	75(52.4)	0.59(0.19–1.81)	0.360		
Occupation						
Worker	38(14.2)	28(19.6)	1.47(0.86–2.52)	0.161		
Farmer	108(40.3)	52(36.4)	0.85(0.56–1.29)	0.459		
Student	21(7.8)	7(4.9)	0.61(0.25–1.46)	0.308		
Cadres	5(1.9)	6(4.2)	2.30(0.69–7.68)	0.202		
Others	97(36.2)	50(35.0)	0.95(0.62–1.45)	0.830		
Residence						
Urban	122(45.5)	54(37.8)	Reference			
Rural	146(54.5)	89(62.2)	1.38(0.91–2.09)	0.143		
Smoking history	94(35.1)	70(49.0)	1.78(1.18–2.68)	0.008		
Excess alcohol consumption	44(16.4)	26(18.2)	1.13(0.66–1.93)	0.680		
Co-morbility						
Hypoalbuminemia	128(47.8)	99(69.2)	2.46(1.60–3.78)	<0.001	1.48(1.18–1.85)	0.001
Anemia	70(26.2)	35(24.6)	0.92(0.58–1.47)	0.812		
Chronic renal failure	2(0.7)	10(7.0)	10.00(2.16–46.29)	0.001	6.67(1.42–31.47)	0.016
Chronic hepatic insufficiency	16(6.0)	28(19.6)	3.84(2.00–7.37)	<0.001	1.99(1.15–3.43)	0.014
Chronic pulmonary disease^a^	41(15.3)	36(25.2)	1.86(1.13–3.08)	0.017		
Gastric ulcer	5(1.9)	8(5.6)	3.12(1.00–9.71)	0.071		
Cardio-cerebrovascular disease	19(7.1)	15(10.5)	1.54(0.76–3.12)	0.261		
Hypertension	21(7.8)	19(13.3)	1.80(0.93–3.48)	0.083		
Diabetes	44(16.4)	22(15.4)	0.93(0.53–1.62)	0.888		
CTD	4(1.5)	9(6.3)	4.43(1.34–14.66)	0.014		
Malignant	2(0.7)	7(4.9)	6.85(1.40–33.40)	0.010		
Invasive mechanical ventilation	3(1.1)	6(4.2)	3.87(0.95–15.71)	0.070		
Central venous catheter	4(1.5)	9(6.3)	4.43(1.34–14.66)	0.014		
Parenteral nutrition	12(4.5)	14(9.8)	2.32(1.04–5.15)	0.053		
MDR-TB	12(4.5)	14(9.8)	2.32(1.04–5.15)	0.053		
XDR-TB	5(1.9)	11(7.7)	5.24(1.37–20.05)	0.019	6.56(1.61–26.69)	0.009
Extrapulmonary TB	53(19.8)	35(24.5)	1.31(0.81–2.14)	0.313		
Hospital LOS (days)	43.00±20.74	60.09±27.50	1.02(1.01–1.04)	0.017		
Duration of TB diagnostic delay (days)	42.6±19.4	57.9±28.3	1.02(1.01–1.03)	<0.001	1.01(1.00–1.02)	<0.001
Hospital mortality	20(7.5)	22(15.4)	2.26(1.19–4.29)	0.016		

Abbreviation: GNB: Gram-negative bacilli; MDR, multidrug-resistant; CTD, connective tissue disease; MDR-TB: multidrug-resistant tuberculosis; XDR-TB, extensively drug-resistant tuberculosis; LOS, length of stay.

Note

^a^ Chronic pulmonary disease included chronic obstructive pulmonary disease, asthma, bronchiectasis.

Univariable comparison showed that the following characteristics predisposed the presence of MDR-GNB: age, smoking history, hypoalbuminemia, chronic renal failure, chronic hepatic insufficiency, chronic pulmonary disease, connective tissue disease, malignant, presence of central venous catheter, presence of XDR-TB, and longer duration of TB diagnostic delay. On the basis of the clinical variables included in univariable comparison, the final multiple logistic regression model predicting MDR-GNB were: age (OR 1.02, 95% CI 1.01–1.03), hypoalbuminemia (OR 1.48, 95% CI 1.18–1.85), chronic renal failure (OR 6.67, 95% CI 1.42–31.47), chronic hepatic insufficiency (OR 1.99, 95% CI 1.15–3.43), presence of XDR-TB (OR 6.56, 95% CI 1.61–26.69), and duration of TB diagnostic delay (OR 1.01, 95% CI 1.00–1.02). The AUC was 0.752 (95% CI 0.715–0.789, P<0.001), and SE was 0.019. The *c* statistic value, which represented by the AUC, was considered acceptable.

## Discussion

To our knowledge, there was no report demonstrating the relationship between XDR-TB and MDR-GNB. The data from three tertiary hospitals in China, showed that the prevalence of patients affected by MDR-GNB in pulmonary TB patients was 4.8% in this study. Patients with XDR-TB had a significantly higher occurrence of MDR-GNB (24.4%) than patients with non-XDR-TB (4.5%). Compared to non-MDR-GNB patients, XDR-TB was an independent risk factor for developing MDR-GNB. Multivariable analysis simultaneously showed that age, hypoalbuminemia, chronic hepatic insufficiency, chronic renal failure, and duration of TB diagnostic delay were the independent predictors of harboring MDR-GNB in pulmonary TB patients.

Patients with XDR-TB are characterized by significant compromised immune balance [2,16] that impacts the patients’ ability to contain other bacterial infection challenges over time. Considering that broad-spectrum antibiotics are frequently used in patients with XDR-TB [9,17], and that broad-spectrum antibiotics have antibacterial activity, XDR-TB patients potentially increase the chance of developing MDR-GNB infection.

Clinical features of TB sometimes are quite similar to those of other bacterial pneumonia at the early stage of the disease, which indicates that the differential diagnosis of TB from bacterial pneumonia is not straightforward. In addition, the etiology cannot be simply differentiated clinically or radiologically. In Asian countries, 1–7% of patients presenting as community-acquired penumonia were rediagnosed as pulmonary TB [18]. The empirical treatment of bacterial penumonia with the regimen of antibiotics can lead to the development of drug resistance [19–21]. XDR-TB patients with longer duration of TB diagnostic delay create favorable conditions for the development of MDR-GNB infection.

In our retrospective study, some patients who developed XDR-TB due to irregular (non-compliant) in taking anti-TB drugs and returned to the clinic with recurrence of symptoms and radiographic progression of the disease. Inadequate treatment can select for the emergence of drug resistant mutations in bacilli [22]. The poor prescribing behavior inherent to the patients could predispose themselves to MDR-GNB infection.

The advanced age, underlying disease, and exposure to antimicrobial drugs are the known risk factors of harboring MDR bacteria [5,10,11,23]. Older individuals tend to have poor immunity defense than younger ones and have a greater likelihood of developing MDR-GNB. Hypoalbuminemia is generally regarded as a marker of poor nutritional status and hypoimmunity [24,25]. Chronic hepatic insufficiency is regularly accompanied by hypoalbuminemia and indicates impaired immunity [26,27], which increases susceptibility to infection with MDR-GNB strains. Patients with chronic renal failure are more likely to develop drug resistant as they are exposed to antibiotics more highly during their clinical courses [28]. Thus more attention should be paid to TB patients with those certain clinical characteristics.

MDR organisms might be associated with either symptomatic illness (infection) or asymptomatic carriage (colonization). Differentiating colonization from infection can be difficult and requires clinical correlation. Given that the majority of colonized patients would have gone undetected, our findings suggest that routine surveillance in patients with XDR-TB may be a beneficial component of MDR-GNB infection control program among TB patients. Delay in appropriate antimicrobial therapy has an adverse influence on the clinical outcome of patients with MDR-GNB. Good communication between the treating clinician and the clinical microbiologist will aid in clinical decision making.

Antimicrobial drug resistance jeopardizes the effectiveness of the treatment of bacterial infections [29]. The emergence and dissemination of MDR-GNB are seriously limiting the options for treatment bacterial infections in TB patients, especially XDR-TB patients. Cases with XDR-TB are virtually untreatable, depending on fewer available medicines [2,9,30]. Since XDR-TB patients have a higher chance of complicating with MDR-GNB, the co-morbidity of two diseases will be the devastating threat to patients. During initiation of new case, proper explanation and completion of the treatment are very important to avoid the development of future drug resistance in the society.

It is noteworthy that about only three-quarters of patients had performed DST in our study. Despite the known high rates of drug resistance TB in China, clinicians often make empirical treatment without laboratory confirmation, partly due to long time to perform DST. Many of the patients without DST may be infected with drug-resistant strains and thus to have been treated with inadequate TB therapy. Undiagnosis of drug-resistant strains will continue to transmit to others, and delay in therapy will result in advanced disease, treatment failure and death [31,32]. All TB optimal treatment regimens should be constructed according to DST results.

Our study has two substantial limitations. First, this study was processed in a retrospective observational manner. The limitation of our study included the relatively small number of XDR-TB patients that were identified. A small data set might introduce sampling bias. Although the conclusions based on these data were statistically significant, a larger data set would strengthen these conclusions. Second, the information about the medication history of antibiotics was limited in the pre-existing database. The main driver of drug resistance is the history of antibiotic use. However, the role of medication history of antibiotics before admission was difficult to track in our retrospective study, as the data were not sufficiently robust due to a high percentage of unknown/unreliable results for self-reporting. The future study would be strengthened by addition of all antibiotic usage data prior to XDR-TB and MDR-GNB diagnosis.

## Conclusions

Since there is a higher occurrence of harboring MDR-GNB among patients with XDR-TB, the judicious use of antimicrobials in TB patients is pivotal. The presence of XDR-TB is an independent risk factor of MDR-GNB in pulmonary TB patients. This work suggests that routine surveillance among XDR-TB patients has a fair sensitivity for identify patients harboring MDR-GNB. Further work is needed to invest the underlying mechanism association between XDR-TB and MDR-GNB.
